# Modulation of Immunity and Inflammation by the Mineralocorticoid Receptor and Aldosterone

**DOI:** 10.1155/2015/652738

**Published:** 2015-09-10

**Authors:** N. Muñoz-Durango, A. Vecchiola, L. M. Gonzalez-Gomez, F. Simon, C. A. Riedel, C. E. Fardella, A. M. Kalergis

**Affiliations:** ^1^Instituto Milenio en Inmunología e Inmunoterapia, Departamento de Genética Molecular y Microbiología, Pontificia Universidad Católica de Chile, Alameda 340, 8331150 Santiago de Chile, Chile; ^2^Instituto Milenio en Inmunología e Inmunoterapia, Departamento de Endocrinología, Pontificia Universidad Católica de Chile, Lira 85, 8330074 Santiago de Chile, Chile; ^3^Instituto Milenio en Inmunología e Inmunoterapia, Departamento de Ciencias Biológicas, Facultad de Medicina, Universidad Andres Bello, República 217, 8370146 Santiago de Chile, Chile; ^4^Departamento de Inmunología Clínica y Reumatología, Facultad de Medicina, Pontificia Universidad Católica de Chile, Alameda 340, 8331150 Santiago de Chile, Chile

## Abstract

The mineralocorticoid receptor (MR) is a ligand dependent transcription factor. MR has been traditionally associated with the control of water and electrolyte homeostasis in order to keep blood pressure through aldosterone activation. However, there is growing evidence indicating that MR expression is not restricted to vascular and renal tissues, as it can be also expressed by cells of the immune system, where it responds to stimulation or antagonism, controlling immune cell function. On the other hand, aldosterone also has been associated with proinflammatory immune effects, such as the release of proinflammatory cytokines, generating oxidative stress and inducing fibrosis. The inflammatory participation of MR and aldosterone in the cardiovascular disease suggests an association with alterations in the immune system. Hypertensive patients show higher levels of proinflammatory mediators that can be modulated by MR antagonism. Although these proinflammatory properties have been observed in other autoimmune and chronic inflammatory diseases, the cellular and molecular mechanisms that mediate these effects remain unknown. Here we review and discuss the scientific work aimed at determining the immunological role of MR and aldosterone in humans, as well as animal models.

## 1. Introduction

Aldosterone is a steroidal hormone produced in the cortex of suprarenal gland that specifically binds to the mineralocorticoid receptor (MR). The production and secretion of this hormone are mainly triggered in response to changes in blood perfusion, which is sensed by principal cells in the juxtaglomerular apparatus [1]. Once aldosterone is produced and secreted, epithelial cells from renal tubule [2] or vascular smooth muscle cells [3] respond by inducing the expression of genes related to water absorption, such as epithelial sodium channel (ENaC), sodium-potassium ATPase, and serum/glucocorticoid regulated kinase 1 (SGK1) [4, 5]. The main goal of these processes is to maintain the body blood pressure in a normal range by means of water and electrolyte homeostasis control. For that reason, aldosterone is also known as a mineralocorticoid (MC) and this physiological network takes place in MC-sensitive tissues that express the MR [1]. Even though aldosterone is the cognate ligand of the MR, glucocorticoids (GCs) such as cortisol can also bind to this receptor with equivalent affinity [6].

Although the effects mediated by aldosterone have been described in renal and vascular tissue, recent reports showed that MR is also expressed in other tissues turning them sensitive to aldosterone stimulation, such as heart [7–9], blood vessels [10], eyes [11, 12], adipose tissue [13, 14], hippocampus [15, 16], and cells of the immune system.

In the context of the immune response, it has been consistently reported that aldosterone stimulation promotes proinflammatory responses in various tissues [17, 18]. In human leucocytes, MR expression has been reported in CD34+ hematopoietic progenitor, also in peripheral blood T and B lymphocytes, monocytes, and neutrophils [19]. Further, clinical studies have demonstrated that MR antagonism in cardiovascular diseases can generate a beneficial outcome in patients, mainly due to the prevention of inflammatory damage [20].

In mice, MR expression has been shown in monocytes/macrophages [21, 22] and dendritic cells (DCs) [23]. On the contrary, expression of this receptor in lymphoid cells remains controversial. In these animals, MR has been mainly studied in hypertension models, demonstrating that its activation in myeloid cells is necessary to develop such a pathology [24]. It is thought that MR modulates the function and activation of macrophages during the development of cardiac fibrosis [25, 26]. Consistent with this notion, macrophages can undergo two types of activation, characterized by differential gene expression programs depending of the triggering stimulus [27]. The first type of activation is known as classical and leads to inflammatory or M1 activated macrophages. These cells are characterized by the secretion of proinflammatory cytokines, the production of reactive oxygen species (ROS), and an enhanced microbicidal and tumoricidal capacity in response to microbial challenges [27]. On the other hand, an alternative or M2 macrophage activation is related with tissue remodeling, wound healing, immune regulatory functions, and fibrosis, as well as with chronic inflammatory conditions. These cells respond to interleukin- (IL-) 4 and IL-13, by inducing the expression of scavenger, mannose and galactose receptors, which confer M2 macrophages with an enhanced phagocytic activity [27]. In addition, molecules such as GC and IL-10 promote a third type of macrophage phenotype that shows overlapping characteristics with M2 macrophages and is known as “M2-like” phenotype [28, 29].

In another type of myeloid cells, dendritic cells (DCs), it was shown that MR stimulation with aldosterone induces the secretion of IL-6 and TGF-*β*, two proinflammatory cytokines able to polarize the adaptive immune response towards a Th17 phenotype that enhances autoimmunity [23]. Furthermore, MR antagonism with spironolactone in a hypertension rat model reduced end-organ damage due to blockade of Th17 polarization and the induction of regulatory T cells [30]. These studies clearly suggest that MR stimulation or antagonism plays a role not only in innate immune activation, but also in the polarization of the adaptive immune response.

The above described data suggest the MR stimulation is important not only for blood pressure control, but also for the modulation of the immune and inflammatory responses. However, up to date, the mechanisms explaining these observations are not completely understood.

## 2. Adrenocortical Hormone/Receptor Pairs with Immunomodulatory Function

The suprarenal cortex produces hormones with different physiological functions: MCs, GCs, and androgens [31]. Although all these hormones display very specific physiological functions, such as electrolyte homeostasis, stress response, carbohydrate metabolism, and sexual development, MCs and GCs have also been strongly associated with the modulation of various cells of the immune system [31].

MR is the nuclear receptor associated with the function of aldosterone, which is encoded by the NR3C2 gene localized in the region q31.1 of chromosome 4. As most nuclear receptors, the MR protein structure is divided into three domains, which include the following: (1) an N-terminal domain (NTD) of 602 aa, involved in the control of the transcriptional activity of the receptor; (2) the central DNA-binding domain (DBD) of 66 aa, responsible of binding to specific Hormonal Response Elements (HREs) found on the promoter of MR target genes; and (3) a hinge region linking them to a C-terminal ligand-binding domain (LBD) of 251 aa responsible for the selectivity of hormone binding [32]. MR belongs to the steroid/thyroid/retinoid superfamily receptors, which are ligand dependent transcription factors [33].

Cortisol was the first adrenocortical hormone described as an immune regulator, specifically as an immunosuppressor [34]. Physiologically, cortisol plays a central role in metabolism, stress responses, and cardiovascular system control [35]. The secretion of this hormone is mainly regulated by an elaborated feedback that includes the hypothalamic-pituitary-adrenal axis (HPA) in order to maintain resting homeostasis [36]. Canonically, GCs act through the activation of GR, which also belongs to steroid/thyroid/retinoid superfamily receptors. In addition, GCs can induce the activation of the MR [34].

Although aldosterone and cortisol differ greatly in their physiologic effects, molecular studies have suggested an apparent paradox as to how these hormones interact and cause a wide variety of effects that are mediated by closely related receptor proteins [37]. Both aldosterone and cortisol bind to a common consensus palindromic DNA sequence, designated originally as a glucocorticoid response element (GRE) [38–41].

The GRE nucleotide composition was first described in a rat hepatoma cell line infected with mammary tumor virus (MTV), which displays binding sites for GCs able to specifically induce viral genome transcription under GRE stimulation with dexamethasone [38, 39]. These GRE sites are located upstream of the MTV long terminal repeat (LTR) sequence and are composed of a family of octanucleotides related to the sequence AGA[A/T]CA(G)[A/T], repeated approximately 9 times within the strongest binding sites in this model [38]. Further research demonstrated that this GRE site was an inverted, imperfect palindrome consisting in 15 bp, where the conserved hexads are separated by 3 nucleotides (e.g., TGTAGAGGATGTTCT) [42, 43]. The GRE structure is not exclusive for GR binding, as it is also common for other nuclear receptors, such as MR, progesterone, and androgen receptors [44]. For that reason, we will refer from now on to Hormonal Response Elements (HREs) rather than GRE.

Upon ligand binding to MR (either to aldosterone or cortisol), the hormone-receptor complex is translocated to the nucleus, where it binds to HREs and modulates gene expression [44], which is known as the genomic pathway. Among the molecular mechanisms described to add specificity to nuclear receptors is the interaction with additional proteins that bind to sequences for nonreceptor factors, located near the HRE, known as composite response elements. At these sites there are binding proteins able to regulate directly and differentially the GR or MR activity, as is the case of Activating Protein 1 (AP-1) [41]. Another regulatory mechanism consists of the recruitment of selective coactivators or corepressors to the promoter, such as eleven-nineteen lysine-rich leukemia (ELL), Fas-associated factor 1 (FAF-1), nuclear receptor corepressor l (NCoRI), and the silencing mediator of retinoid and thyroid hormone receptor (SMRT) [45]. Additional specificity in favor of aldosterone is related to the higher binding stability of the aldosterone-MR complex, as compared to the cortisol-MR complex [46]. The main cellular mechanism of regulation is the expression 11-*β*-hydroxysteroid-dehydrogenase type 2 (11*β*-HSD2), an enzyme capable of catalyzing the transformation of cortisol into inactive cortisone, reducing the probability of GCs to occupy available MRs [47, 48].

**Figure 1:**
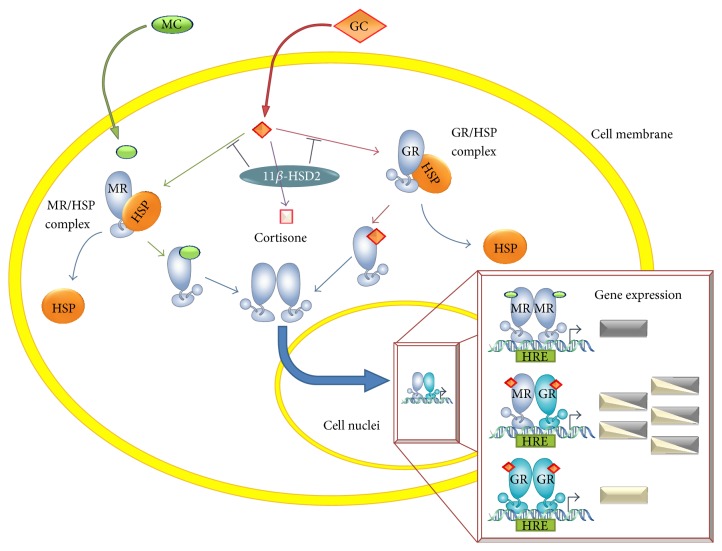
Corticoid signal transduction. Circulating corticoids diffuse across the cell membrane. While mineralocorticoids (MC) almost exclusively bind to the mineralocorticoid receptor (MR), glucocorticoids (GC) can bind either to MR or to the glucocorticoid receptor (GR). GC binding to its receptors is modulated by 11-*β* hydroxysteroid dehydrogenase type 2 (11*β*-HSD2), by converting GCs to its inactive form, cortisone. Upon ligand binding, these nuclear receptors uncouple from their complex with Heat Shock Proteins (HSPs), dimerize, and translocate to the nucleus. Depending on the bound ligands nature, GR and MR can either homodimerize or heterodimerize. These dimers then bind to Hormonal Response Elements (HREs) on promoter regions and different genes will be transcribed.

The MR can form either homodimers or heterodimers with the GR (Figure 1) [49, 50]. Experiments made in the human neuronal cell line BE(2)C that only expresses MR (not GR) showed that aldosterone stimulation only induces the expression of the reporter GRE-luciferase when cells are cotransfected with a GR expression vector [51]. It was also demonstrated that GR-MR works as a specific cooperative complex to promote the binding to HRE, because cotransfection with other steroid receptor failed to show changes in HRE-luciferase expression [51]. Another study performed in a neuroblastoma cell line cotransfected with HRE-luciferase and with expression vectors to GR and MR demonstrated that MR-GR dimers work synergically in response to low doses of cortisol enhancing reporter expression [50, 52]. It was also shown that MR-GR heterodimer formation can take place in a monkey kidney cell line but without the synergic effect described above [43, 51]. The opposite and diverse results described could be due to the different cell types used for the experimentation, to the different cortisol concentrations used, or to the different availability of coactivators or corepressors in each cell line, which would modulate receptors DNA-binding affinity [50].

It has been recently reported that the MR can undergo post-translational modifications that could allow a fine tuning of MR signaling, depending on the physiological context [53]. For example, 14 of 16 phosphorylation sites located in NTD are targets for ERK1/2, cyclin-dependent kinase 5 (CDK5), protein kinase A (PKA), and casein kinase 1. The final effect of these modifications includes the modulation of MR nuclear translocation [54, 55] and ligand binding [56], which impacts the transcriptional activity. Also, the rapid or early effects mediated by aldosterone stimulation induces the nongenomic pathway, related with the regulation of second-messenger systems and phosphorylation of kinases responsible of signal transduction, such as ERK1/2 and c-Jun NH2-terminal kinases 1 and 2 (JNK) [57, 58]. In smooth muscle and endothelial cell cultures, the nongenomic pathway includes modification of inositol-1,4,5-triphosphate and diacylglycerol production and PKC pathway stimulation and increases the intracellular concentrations of calcium and cAMP [59]. In addition, the ubiquitylation of MR is mainly associated with the maintenance of homeostatic levels of MR in the cytoplasm and the transition between unbound to hormone-bound state, because chaperone protein degradation is achieved via the proteasome [60]. Further, MR oxidation in sulfhydryl groups located in the LBD can determine whether ligand binding takes place or not [61, 62]. Recently it has been described that additional modifications, such as acetylation and sumoylation, are related to the control of MR binding to HREs and the recruitment of coactivator molecules [53].

These data suggest that aldosterone, cortisol, MR, and GR display very complex ways of interaction, increasing the functional diversity of these hormones in diverse biological systems.

## 3. Clinical Evidence Associating the MR with the Modulation of the Immune System

Currently, the physiopathology of cardiovascular diseases, such atherosclerosis [63] and hypertension, are considered to be closely related with inflammatory processes [64, 65]. As a result, inflammatory cells infiltrate the tissues producing proinflammatory cytokines and inducing the generation of oxidative stress, which leads to fibrosis and end-organ damage [18]. The Renin-Angiotensin-Aldosterone System (RAAS) has been closely related with the deleterious effect seen in patients with cardiovascular diseases, turning RAAS into an important axis that could be intervened to reduce disease [17, 18].

Consistently, two large clinical trials, Randomized Aldactone Evaluation Study (RALES) [66] and Eplerenone (a highly selective MR antagonist) Postacute myocardial infarction Heart and Survival Study (EPHESUS) [67], have shown that MR antagonism, in addition to standard therapy to control hypertension (i.e., angiotensin converting enzyme inhibitors), substantially reduces morbidity and mortality in patients with severe heart failure or with acute myocardial infarction complicated by left ventricular dysfunction and heart failure, respectively. Later, a substudy made in the EPHESUS cohort demonstrated that the positive effects seen in these patients are beyond the diuretic effects and potassium sparing and could be related with an unknown mechanism [20]. Another study made in patients suffering different degrees of heart failure and treated with spironolactone (a potent but not so selective MR antagonist) during 1–3 months showed no effect in lowering serum levels of other inflammatory markers such as C-reactive protein [68]. Not only has the positive effect of MR antagonism been evaluated in patients with cardiovascular diseases, it has also been tested in autoimmune pathologies. For example, 76% rheumatoid arthritis (RA) patients treated during two weeks with spironolactone plus the individual treatment responded favorably to therapy according to American College of Rheumatology criteria, mainly due to lower systemic levels of TNF-*α*, IL-6, GM-CSF, and IFN-*γ* after the treatment [69]. Furthermore, RA patients reported that synovial cells presented abnormally high levels of 11*β*-HSD2 enzyme, which correlated with the level of disease activity [70, 71]. We suggest that this phenomenon would promote the selective binding of aldosterone to MR impairing in this tissue the anti-inflammatory effect of corticosteroid treatment, because this enzyme metabolizes active cortisol into cortisone, which fails to activate GR. This result underscores the complex regulation of MR activation, because it not only is dependent on the ligand, but also could be indirectly stimulated due to 11*β*-HSD2 expression.

Conversely, studies made in types I and II diabetic patients have not shown reduction in serum proinflammatory cytokines and markers of endothelial dysfunction, but an important reduction in urinary albumin excretion and microalbuminuria suggesting that spironolactone confers renal protection in diabetic individuals [72, 73].

## 4. MR Activation and Antagonism in Pathological Conditions

The current literature associates MR activation by aldosterone with the promotion of inflammation and fibrosis, but most of these studies were focussed on hypertension development or the production of inflammatory mediators. Although these animal models usually show increased levels of circulating aldosterone [74], it is important to highlight that GCs can also induce hypertension and tissue damage due to MR activation, because deleterious effects were avoided only when MR antagonists were used, rather than GR antagonists [75, 76]. In conclusion, the inflammatory profile is directly linked to MR activation. In addition, there is evidence suggesting that MR activation also associates with other pathologies, such as autoimmunity, chronic renal disease, and obesity.

### 4.1. Pharmacological MR Antagonism:* In Vitro* Assays

Two generations of MR antagonists have been developed. The first generation included spironolactone and canrenone, two potent steroidal compounds that are also androgen receptor antagonists and progesterone receptor agonists and produce several active metabolites [77]. More recently, a new steroidal MR antagonist was developed: eplerenone, which is less potent than the first generation and has a shorter half-life but is more selective than the previous compounds for the MR and generates no other active metabolites [77].

Based on the observations related with the anti-inflammatory and antifibrotic role of MR antagonism in patients with congestive heart failure, studies were carried out to dissect the mechanism behind it.* In vitro* studies made in healthy human peripheral blood mononuclear cells (PBMCs) stimulated with spironolactone showed an immune-modulatory effect* per se*, in which proinflammatory cytokines related with Th1 immune response (TNF-*α* and IFN-*γ*) were decreased in contrast to Th2 and anti-inflammatory cytokine expression and production [69, 78]. Another study showed that spironolactone can modulate the function of other transcription factors that control the immune response, such as NF*κ*B, CEBP*β*, and MYC [79]. Along these lines, experiments made in human monocytes treated with eplerenone showed that MR antagonism helps to modulate the type of macrophage activation, turning those cells into an alternative activation phenotype [80]. Although these studies clearly associate MR antagonism with anti-inflammatory effects, neither of them showed changes in IL-1*β* expression. These data suggest that MR antagonism does not inhibit the inflammasome pathway [69, 79], or the production of cytokines related with alternative macrophage polarization, including IL-4 or the anti-inflammatory cytokines TGF-*β* and IL-10 [25, 69]. In contrast, it has been described that treatment of peritoneal macrophages with low doses of corticosterone (10 nM) increases the expression of* Tnfα* and this proinflammatory cytokine only returns to basal levels when GR but not MR is blocked [81].

**Figure 2:**
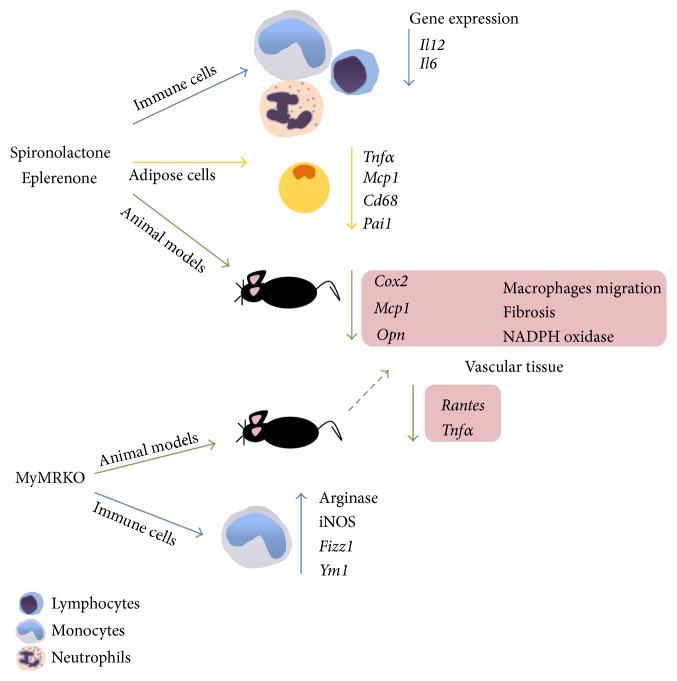
Immunological effect of MR antagonism and knockout.* In vitro* treatment with pharmacological antagonist of MR has suggested that this receptor could be important to keep immune function, because its antagonism directly modulates the proinflammatory cytokines and chemokines production and cell migration. These results also have been reported in other cell types such as adipocytes, which today are known as a source of inflammatory mediators. In animal models the treatment with MR antagonist or KO animals to myeloid MR has supported the notion of MR as immune regulator.

During obesity and metabolic syndrome an inflammatory state is established due to the production of proinflammatory cytokines, chemokines, and prothrombotic factors by the adipose tissue [82]. Consistently, it has been observed that MR antagonism* in vivo *can reverse the expression of obesity-related inflammatory genes in adipose tissue, such as* Tnfα*,* Mcp1*,* Cd68*, and plasminogen activator inhibitor-1 (*Pai1*) and promote the expression peroxisome-proliferator activated receptor gamma (*Pparγ*) and adiponectin [83, 84]. Conversely,* in vitro* studies in brown fat showed that aldosterone stimulation can inhibit the expression of uncoupling protein 1 and promote leptin and* Mcp1* adipokine expression and stimulate insulin resistance [85] (Figure 2). Further, studies using 3T3-L1 preadipocytes cell line treated with aldosterone or aldosterone plus canrenoate (MR antagonist) showed the same results as seen* in vivo*, suggesting that MR stimulation by aldosterone may have a local, proinflammatory role in adipose tissue [83].

Moreover, components of the RAAS are produced by adipocytes [86]. In addition to angiotensin II (Ang II), adipocytes secrete mineralocorticoid releasing factors that stimulate steroidogenesis in human adrenocortical cells [87–90] which might explain the hyperaldosteronism often observed in obese subjects. A recent report also supports the hypothesis that adipocytes are able to produce and secrete modest amounts of aldosterone, which may contribute to total circulating aldosterone levels and the higher degree of MR activation in obesity [91]. Moreover, there is evidence of an adipocyte-derived factor that stimulates the adrenal gland to generate excessive aldosterone synthesis [90, 92]. Both the cause of this high aldosterone level and the adipocyte-derived factors identity remain unknown. Nevertheless, there is evidence of the adiponectin receptor being expressed in both the human adrenal cortex and aldosterone-producing adenomas and recently and also of adipose tissue being capable of directly synthesizing aldosterone [91, 93].

### 4.2. *In Vivo* Pharmacological Antagonism of MR and Knockout Models

Studies performed in hypertensive animal models with pharmacological MR antagonists (spironolactone or eplerenone) have revealed that these drugs protect against inflammation development and end-organ damage, rather than preventing the occurrence of hypertension. Saline-drinking stroke-prone spontaneously hypertensive rats (SHRSP) treated with spironolactone showed no improvement in blood systolic blood pressure but displayed cerebrovascular and renal protection, measured as reduced urinary protein excretion and histopathology analyses [94]. The use of eplerenone in another model of hypertension, in which aldosterone-salt is administrated in uninephrectomized rats, slightly diminished systolic blood pressure, but the effect was not completely abrogated as compared with sham rats [21]. However, histological analyses showed that monocyte/macrophage infiltration in vascular wall induced by aldosterone-salt was abolished in eplerenone treated rats. In addition, gene expression of proinflammatory molecules, such as* Cox2*,* Mcp1*, and* osteopontin (Opn)* was dramatically diminished in this group as compared to nontreated rats [21]. Along these lines, specific MR antagonism by eplerenone not only prevented vascular wall inflammation and fibrosis, but also reversed an initially established inflammation and fibrosis developed in kidney and cardiac tissues, mainly due to higher expression of genes related with the NADPH oxidase system [22, 95]. Further, animals treated only with eplerenone after renal interstitial injury presented renal protection seen as reduced fibrosis changes and inflammatory infiltration. Independently of the aldosterone presence, the above data suggest a striking and not completely understood relationship between MR antagonism and tissue protection, with an important participation of immune cells. These results also underscore that specific MR antagonism generates similar beneficial outcomes, independently of the model used to induce inflammatory renal tissue damage.

Subsequent studies made in a hypertensive mouse model mediated by deoxycorticosterone acetate- (DOCA-) salt in wild-type mice showed an increase in blood pressure in the chronic phase of the disease (4–8 weeks) and in the percentage of collagen deposition in perivascular and interstitial heart wall [26]. The opposite result was obtained in conditional MR knockout in myeloid cells of immune system (MyMRKO) generated by Cre/LoxP system [26]. These differences can be explained neither by changes in macrophage recruitment to vascular wall, which could explain the differences seen in antifibrotic phenotype, nor by different expression in MCP-1. Another analysis of gene expression* in situ* showed low baseline expression of some proinflammatory genes and profibrotic factors, such as* Opn*,* Col1*,* Tgfβ1*, and* Pai1*, which suggested a loss of classical macrophage activation [26]. Primary cultures of murine peritoneal macrophages from MyMRKO showed that loss of MR activation drives these cells towards an alternative activation, measured by gene expression [25].* In vivo* experiments made in MyMRKO mice, but in the hypertensive model mediated by angiotensin II/L-NAME, did not show protection to develop hypertension but presented reduced signs of aortic fibrosis and hypertrophy. These findings in MyMRKO mice were related with low macrophage recruitment to vascular wall and lower gene expression* in situ* of* Rantes* and* Tnfα*, compared with litter mate mice [93].

In the brain, MR activation is important and necessary to maintain many of the functions of this tissue. Under pathological conditions, such as cerebral ischemic stroke model by middle artery cerebral occlusion, MR antagonism has been associated with a reduction of ischemic area and also lower markers of remodeling process in cerebral vessels, including changes in wall thickness suggesting that this phenomenon could be mostly due to a reduction in superoxide production [96]. These results also have been seen in MyMRKO mice, which also showed reduced ischemic cerebral areas as compared to control animals [97]. In the MyMRKO model a reduction of microglial/macrophages infiltration and activation in the infracted zone was also reported, which presented a lower expression of inflammatory cytokines and chemokines, such as* Il1b*,* Il6*,* Tnfα*,* Mcp1*, and* Mip1*, but did not show differences in the expression of markers of alternative macrophage activation, including inducible nitric oxide synthase (iNOS) and arginase [97] (Figure 2).

Therefore a role for MR in the modulation of immune cell function under homeostatic and pathologic condition is supported by extensive literature. The available data are related to macrophage function, suggesting that MR expression is important to determine the phenotype of activation of these cells during hypertension, stroke, obesity, and autoimmunity, leading to the proposal of MR as an important pharmacological target for preventing chronic inflammatory conditions.

## 5. Aldosterone and the Inflammatory Response

### 5.1. Lessons from Primary Aldosteronism

Primary aldosteronism (PA) is a condition with abnormally high levels of aldosterone that in 95% of the cases is due to unilateral adenoma and bilateral idiopathic adrenal hyperplasia and causes excessive MR stimulation [98]. Since PA first description of an aldosterone-producing adrenal adenoma, the excessive aldosterone production and the pathophysiological mechanisms of hypertension have been a research challenge [99]. Follow-up studies on these patients after tumor removal have shown normalization or significant reduction of their blood pressure [100, 101]. Currently it is thought that PA is the cause of secondary hypertension in approximately 5–10% of hypertensive patients [102, 103]. Further, it has been reported that these patients show worsened symptoms of systemic inflammation [104, 105] and renal tissue damage [106], as compared to essential hypertensive (EH) patients [107]. As PA patients also present more cardiovascular complications, such as left ventricular hypertrophy [108, 109], stroke, nonfatal myocardial infarction, and atrial fibrillation [105], the increase in the aldosterone-renin ratio in resistant hypertension patients has been used as a predictor of exacerbated cardiovascular injury and increases the risk of developing uncontrolled resistant hypertension [110].

Specifically, renal damage mediated by aldosterone is characterized by proteinuria, collagen accumulation, glomerular structural lesions, and microalbuminuria [111–113]. These deleterious effects of aldosterone on kidney function appear to be due in part to the production of reactive oxygen species (ROS) [114], which activate the mitogen-activated protein kinases (MAPK) pathway in renal cortical tissues, triggering renal injury [114]. Accordingly, serological measurements have shown that PA patients display elevated levels of malondialdehyde, a lipoperoxidative marker of endothelial inflammation related to oxidative stress, and the aminoterminal propeptide type I (PINP-1), a marker of myocardial collagen synthesis, as compared to EH patients [104, 115]. Both markers decreased after specific treatment of PA. Further, in animal models aldosterone has shown inducing an imbalance in the production of ROS and reactive nitric species [116]. Measurements of superoxide production in the arterial wall [117], kidney [118], and brain [119] of DOCA-hypertensive rats showed a significant increase in superoxide production in these tissues as compared to untreated animals. On the contrary, inhibition of ROS production or MR activation ameliorated hypertension development and other detrimental side effects in all tissues. Complementary studies made in mice deficient for gp91phox or p47phox, subunits of the NADPH oxidase system, showed a partial attenuation of the hypertension development induced by DOCA/salt at an early time point [120].

Beyond ROS involvement in the hypertensive phenotype and tissue damage, it has been demonstrated that aldosterone and its oxidative properties can directly damage DNA and contribute to future cancer development [118, 121]. Experiments made in kidney tubular cell lines demonstrated that aldosterone induces DNA damage via MR, measured by TUNEL assay and micronucleus formation [121]. This phenomenon was prevented using MR antagonism and antioxidant substances, such as N-acetyl cysteine and *α*-lipoid acid, suggesting that oxidation through MR activation is necessary to induce DNA damage. Furthermore, this notion was supported by* in vivo* experiments in DOCA/salt hypertensive rats, which showed a higher renal DNA damage in contrast with sham rats or MR antagonism [118].

In addition, high plasma levels of aldosterone can induce structural and functional alterations in the heart, kidneys, and blood vessels, such as vascular inflammation, cardiac fibrosis, nephrosclerosis, and tissue remodeling [122–125]. These effects may be a consequence of direct actions of aldosterone on fibroblasts and vascular cells. Furthermore, the effects of aldosterone over cardiac myocyte were suggested when an excess of aldosterone in a high dietary Sodium context was observed, which could be associated with cardiac myocyte necrosis [126].

### 5.2. Linking Inflammation to Aldosterone

**Figure 3:**
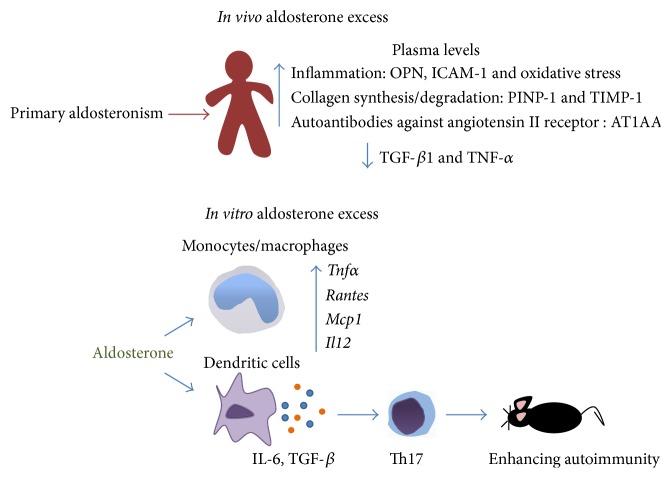
Aldosterone as proinflammatory stimulus. Available data links aldosterone excess with inflammatory phenotype. Clinical studies performed in patients with primary aldosteronism have demonstrated that aldosterone induces inflammatory and oxidative changes, whereas MR antagonism or controlling aldosterone levels contributes to return at homeostatic conditions. Similarly,* in vitro* experiments demonstrate that myeloid immune cells respond to aldosterone, inducing the expression and secretion of proinflammatory cytokines. This modulation of innate immune cells directly impacts the polarization of adaptive immune response toward Th17 phenotype.

A recent study in patients suffering from aldosterone-producing adenomas and showing resistant arterial hypertension reported that they secreted significantly high levels of TNF-*α*, IL-6, and IL-1*β* from monocytes and IL-2, IFN-*γ*, and TNF-*α* from lymphocytes secreted at the beginning of the study. Two months after spironolactone, eplerenone, or adrenalectomization therapy, the levels of all of cytokines were significantly decreased during the 24 months of the clinical study down to levels equivalent to EH and healthy controls [127]. Another study described higher levels of OPN in PA patients compared to EH patients, with no changes between groups in other markers of systemic inflammation, such as IL-6, TNF-*α*, and CRP [128]. Further, we reported that PA patients displayed increased serum levels of TNF-*α* and IL-10, as well as lower serum levels of TGF-*β*1 as compared to EH patients. Spironolactone treatment of PA patients restored serum levels of all three cytokines [129]. In addition, PA patients and preeclamptic women showed higher titers of autoantibodies against angiotensin II receptor 1 (AT1AA) in serum, as compared to EH patients, normotensive and normal pregnancy women [130, 131]. Also, AT1AA showed agonistic activity that stimulates aldosterone secretion in HAC15 adrenal cells [130, 131] suggesting that these antibodies may also be contributing to the vascular and renal tissue damage seen in these patients. All of these data clearly associate aldosterone excess with proinflammatory phenotype (Figure 3).

*In vitro *studies directed to understand the mechanism by which aldosterone induces an inflammatory phenotype have been made in isolated peritoneal macrophages from wild-type mice. After aldosterone treatment, these cells displayed a “classical” activation macrophage phenotype, in which* Tnfα*,* Rantes*,* Mcp1*, and* Il12* expression was induced, which was prevented after eplerenone pretreatment, indicating that proinflammatory cytokine expression was through MR [25]. In addition, we recently demonstrated that murine dendritic cells express MR and respond to aldosterone stimulation by inducing the secretion of IL-6 and TGF-*β* [23]. Both cytokines induced T cell polarization towards Th17 phenotype and enhance autoimmune damage in an* in vivo* model of experimental autoimmune encephalomyelitis [23] (Figure 3). In the same line, the balance between Th17 pathogenic and T regulatory cells during hypertension mediated by DOCA-salt in rats is altered. A lower ratio of Th17/Treg is seen in PBMCs, kidney, and heart of hypertensive rats; however the Th17/Treg ratio could be restored in animals that received spironolactone treatment [30].

Despite wide evidence of MR activation being proinflammatory, controversial data was recently published in eye tissue, which is considered a MC-sensitive organ. In a mouse model of endotoxin-induced uveitis, only the concomitant administration of aldosterone and LPS reduced the intensity of clinical inflammation, the release of inflammatory cytokines such as TNF-*α*, IFN-*γ*, and MIP-1*α* at early times, and the number of activated microglia/macrophages in this tissue. Together with these results, shortly after endotoxin-induced uveitis onset, 11*β*-HSD2 expression was downregulated in iris/ciliary body, and corticosterone levels were increased in aqueous humor [12]. From these results we postulate that considering that 11*β*-HSD2 expression is reduced the anti-inflammatory effect of aldosterone seen in this tissue cannot be strictly explained by its binding to MR, because both GCs levels and GRs expression were increased.

It has been suggested that aldosterone, through MR, could be regulating the expression of several genes involved in vascular fibrosis, calcification, and inflammatory damage to human vascular smooth muscle cells (VSMCs) [3, 59, 132, 133]. More recently, it was shown that VSMCs express intercellular adhesion molecule- (ICAM-) 1 in response to aldosterone via MR, which promotes leukocyte adhesion by specific association with lymphocyte function-associated antigen- (LFA-) 1 expressed on the leukocyte surface [134]. Moreover, there is evidence suggesting that aldosterone can be synthesized directly by heart tissue and possibly has local effects through autocrine/paracrine pathways [135, 136]. However, this notion remains controversial [137, 138].

To understand the molecular mechanism by which MR carries out its proinflammatory activity, we must take advantage of the knowledge about the way GCs act as immunosuppressor: controlling the gene expression of proinflammatory cytokines. The cytokine promoters contain HREs, which can be modulated by GCs and potentially by MCs [139]. It was described that ligand-receptor binding to simple HRE is enough to induce gene expression [41]. However,* in vivo* there are many other proteins able to modulate these interactions to add specificity. Also, this interaction could be more complex when the HRE also contains binding sites for nonreceptor factors, for example, composite HREs. Experiments made in a composite HRE reporter construct, pIFG3, demonstrated that AP-1 proteins (cFos and cJun) are essential to control genomic regulation of GR [41]. Opposite results were obtained when MR was tested in the same experimental conditions, leading to the conclusion that in the same composite HREs only GR but not MR function is directly modulated by AP-1 proteins [41]. During inflammatory responses, large amounts of proinflammatory signals are produced that are transduced through intracellular signaling pathways [139]. One of these pathways is the MAPKs, belonging to JNKs and ERKs that phosphorylate c-Jun and c-Fos proteins, respectively [140]. As we noted before, only GR but not MR is negatively modulated by cFos and cJun; hence it is possible that this phenomenon helps MR binding to the HRE in the promoters of inflammatory genes and inducing gene expression upon inflammatory stimulation.

Studies made in nonimmune cells, such as mesangial cells, have demonstrated that MR stimulated by aldosterone induces the expression of* Opn*, due to the existence of a putative steroid binding site in the promoter at −1984 bp upstream of the coding sequence [141]. Further, it was observed that* Opn* expression was enhanced by the addition of IL-1*β* and TNF-*α* (simulating an inflammatory milieu) and that this effect was completely abrogated by spironolactone addition [141]. A study made in rat renal fibroblast showed that osteopontin promoter also contains binding sites for NF*κ*B and AP-1 proteins and that mutations in these sites can abolish the promoter activity induced by aldosterone stimulus [142]. Similar results were corroborated in VSMCs of rats, also informing the specific location of the HRE in the OPN promoter and the consensus sequence of the MR binding site [143].

Along these lines, a study made in microglial cells BV-2 reported that selective stimulation with aldosterone exclusively resulted in NF-*κ*B activation and the nuclear translocation of its subunit p65, further upregulating the expression of* Il6* and* Tnfr2*, whereas dexamethasone treatment had opposite effects [144]. Also chromatin immunoprecipitation experiments made in cardiomyocytes demonstrated that the MR binds on the neutrophil gelatinase-associated lipocalin (NGAL) promoter and, furthermore, aldosterone treatment induced* Ngal* expression [145]. This observation suggests the possibility of the MR being a pathologic modulator of* Ngal* expression and further NGAL/matrix metalloproteinase 9 complex mediated tissue damage in an atherosclerotic plaque model [146].

Up to date there are no available data about the direct molecular mechanisms by which the MR is interacting with cytokine promoters in immune cells, despite the large amount of evidence that links MR/aldosterone with a proinflammatory phenotype. However, based on the data discussed above, it seems likely that the MR/aldosterone effect in immune cells could be mediated not only by the presence of HREs in cytokine promoters, but also by the presence of AP-1 sequences that allows MR binding without being modulated by AP-1 proteins [41].

## 6. Concluding Remarks

The effect of aldosterone or MR activation has been studied separately for many years, principally in the context of physiological response in cardiovascular and renal tissue. Once reporting that MR is expressed in extravascular tissues, different models were developed to study the effects of aldosterone excess and MR activation, and large amount of data has related RAAS axis with inflammatory phenotypes. Other works supported the idea of aldosterone as inflammatory molecule, because MR antagonism promotes anti-inflammatory phenotypes. MR antagonism results described in human patients and in* in vitro* experiments suggest that the immune axis affected by aldosterone stimulation is very specific, affecting mainly the proinflammatory and pathogenic Th1/Th17 polarization and not generating changes in either inflammasome pathway or IL-10 and CRP secretion. These results suggest that MR antagonism could be beneficial in chronic pathologies with Th1/Th17 phenotype.

The specificity of aldosterone-MR binding is regulated by cortisol levels and 11*β*-HSD2 enzyme expression. Excessive cortisol that cannot be metabolized because of 11*β*-HSD2 absence will favor GC binding to MR, thus generating a different transcriptional profile than the expected upon aldosterone stimulation.

11*β*-HSD2 brings order to the complexity of the possible interactions between GR and MR binding to GCs or MCs previously seen. This enzyme will define the specificity of the response favoring the formation of MR-MC complex over MR-GC complex. Thus, it will orchestrate whether MR-MR or GR-GR and GR-MR dimers are formed and, furthermore, determine target gene expressions.

MR has been shown to be essential for life, since MR knockout mice are nonviable [147]. We have presented evidence of MR not only being essential in renal and vascular tissues, but affecting the functionality of diverse tissues and, more importantly, we underscore the importance of MR and aldosterone stimulation in the immune system.
